# When You Were a Tadpole and I Was a Fish

**DOI:** 10.3201/eid1504.000000

**Published:** 2009-04

**Authors:** Polyxeni Potter

**Affiliations:** Centers for Disease Control and Prevention, Atlanta, Georgia, USA

**Keywords:** Art science connection, emerging infectious diseases, art and medicine, Amazon River, fish, dolphin, Ray Troll, Fishes of Amazonia, about the cover

**Figure:**
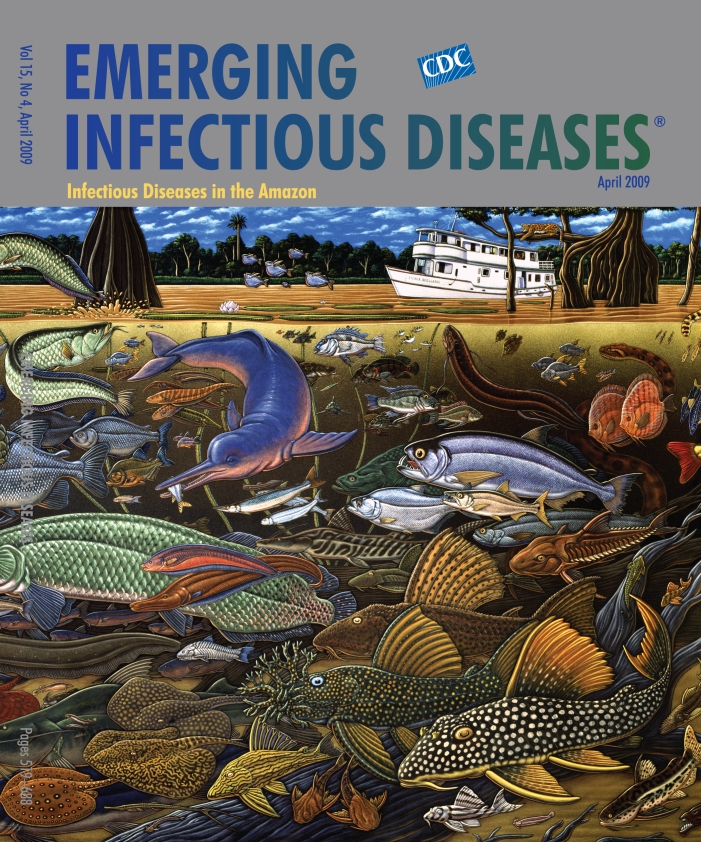
**Ray Troll (b. 1954) Fishes of Amazonia (2000)** Acrylic on canvas (21.13 m × 4.57 m) Miami Museum of Science, Miami, Florida, USA

“All of us with backbones are … the fish group,” realized Ray Troll somewhere in his understanding of evolutionary biology. Our hands are but an evolved version of fins. When you know this, “Then you start seeing the world in a different way.” This evolutionary connection fueled the artist’s imagination as well as his interest in the natural world and its origins, which rivals only his compulsion to paint.

Troll was born in Corning, New York, and spent his early years at various locations where his family lived. He studied art at Bethany College, Kansas, and then moved to Seattle to continue his studies at Washington State University. In 1983, he went to Alaska to visit his sister, “Come on up and visit Ketchikan …,” she had written, “Why don’t you work up here for the summer?” Enthralled with the beauty of the environment and the pace of the local community, he made this city between a rainforest and the salty water of the Tongass Narrows his home. He incorporated it in his unique style, a bold mixture of naturalist imagery and eccentric humor reminiscent of Brueghel or Bosch that captivates scientist and artist alike.

“At first I didn’t notice it,” Troll said of indigenous art, another major influence on his development. “But then I fell in love with it.” During his travels in the area, he mixed with artists from the coastal clans and watched them work with wood and make totem poles. His iconic representations of fish, which have earned him honorary membership in the Gilbert Ichthyological Society and the Guild of Natural Science Illustrators, reflect the linearity of Northwest Native American design.

A disciplined artist, Troll works out of his waterfront studio, which is filled with fish specimens, fossils, and paleontology texts, “I like to let the art take its time.” He travels around the country looking for dinosaur bones and new fish. His drawings and paintings fill science books, anchor multimedia exhibits, or appear online to aid species identification for the National Marine Fisheries Services. One of his murals graces The National Oceanic and Atmospheric Administration Fisheries Laboratory in Santa Cruz. And *Hydrolagus trolli*, a species of ratfish, was named after him.

After an autumn 1997 trip down the Amazon River, some 1,600 km, with friends and scientists, Troll produced a mural-sized painting that took a year to complete: Fishes of Amazonia, on this month’s cover. The river, home to more than 2,000 known fish species (a fraction of its full inventory), inspired this artistic celebration. Species diversity, due only partly to the river’s immense size and abundance of light, food, and favorable temperatures, may have come about from climatic and geographic shifts over millions of years. During these massive changes, species appeared and disappeared, and populations evolved as they adapted to new conditions. Fish recount the long history of the earth.

“The salmon-falls, the mackerel-crowded seas, / Fish, flesh, or fowl, commend all summer long / Whatever is begotten, born, and dies,” wrote W.B. Yeats in “Sailing to Byzantium.” Awed by the vastness of the sea and the bounty of the world but troubled by their transience, the poet saw permanence only in art as in Byzantium. But the study and recording of species for posterity, left to the scientist and artist, betray a remarkable continuity unseen by the poet. For Troll, these disciplines converge to explore the connection between species diversity and the evolutionary changes that produced it, a connection also pertinent on the microbial level.

Emerging infections are often caused by pathogens present in the environment but brought out of obscurity and given a selective advantage or the opportunity to infect new populations by changing conditions. The presence of malaria, dengue fever, orally transmitted Chagas disease, Kaposi sarcoma–associated herpesvirus infection, adiaspiromycosis, and many other emerging diseases indicates that emergence and epidemiologic change are vigorous and ongoing in the Amazon Basin.

The Amazon River dolphin, adapted for life in turbid waters, has tiny eyes and uses instead advanced echolocation to find prey. Along the same evolutionary lines, the presence of lobomycosis in these dolphins poses the likelihood that the agent of the disease has found ways to infect a broader array of the species than we know in the same ecologic community.

British naturalist Langdon W. Smith in his poem A Tadpole and a Fish (or Evolution, 1909), traced life back to the Paleozoic era, the beginning of life. Threading changes back and forth, between darkness and light, throughout the eons, with humor and romance, he arrived in our times. “Then as we linger at luncheon here, / O’er many a dainty dish,” he wrote warning us to be mindful of our origins, “Let us drink anew to the time when you / Were a Tadpole and I was a Fish.”
